# Methicillin resistant *Staphylococcus aureus* in Ethiopia: a meta-analysis

**DOI:** 10.1186/s12879-016-2014-0

**Published:** 2016-11-21

**Authors:** Setegn Eshetie, Fentahun Tarekegn, Feleke Moges, Anteneh Amsalu, Wubet Birhan, Kahsay Huruy

**Affiliations:** 1 Department of Medical Microbiology, College of Medicine and Health Sciences, University of Gondar, P. O. Box: 196, Gondar, Ethiopia; 2 Department of Anesthesia, College of Medicine and Health Sciences, Bahirdar University, Bahirdar, Ethiopia

**Keywords:** Methicillin resistant *Staphylococcus aureus*, Meta-analysis, Systematic review, Ethiopia

## Abstract

**Background:**

The burden of methicillin resistant *Staphylococcus aureus* is a major public health concern worldwide; however the overall epidemiology of multidrug resistant strains is neither coordinated nor harmonized, particularly in developing countries including Ethiopia. Therefore, the aim of this meta-analysis was to assess the burden of methicillin resistant *Staphylococcos aureus* and its antibiotic resistance pattern in Ethiopia at large.

**Methods:**

PubMed, Google Scholar, and lancet databases were searched and a total of 20 studies have been selected for meta-analysis. Six authors have independently extracts data on the prevalence of methicillin resistant *Staphylococcus aureus* among clinical isolates of *Staphylococcus aureus*. Statistical analysis was achieved by using Open meta-analyst (version 3.13) and Comprehensive meta-analysis (version 3.3) softwares. The overall prevalence of methicillin resistant *Staphylococcus aureus* and its antibiotic resistance pattern were pooled by using the forest plot, table and figure with 95% CI.

**Results:**

The pooled prevalence of methicillin resistant *Staphylococcus aureus* was 32.5% (95% CI, 24.1 to 40.9%). Moreover, methicillin resistant *Staphylococcus aureus* strains were found to be highly resistant to penicillin, ampicillin, erythromycin, and amoxicillin, with a pooled resistance ratio of 99.1, 98.1, 97.2 and 97.1%, respectively. On the other hand, comparably low levels of resistance ratio were noted to vancomycin, 5.3%.

**Conclusion:**

The overall burden of methicillin resistant *Staphylococcus aureus* is considerably high, besides these strains showed extreme resistance to penicillin, ampicillin, erythromycin and amoxicillin. In principle, appropriate use of antibiotics, applying safety precautions are the key to reduce the spread of multidrug resistant strains, methicillin resistant *Staphylococcus aureus* in particular.

## Background

*Staphylococcus aureus* is one of a versatile pathogen and the main cause of hospital and community acquired infections, the disease ranging from mild skin infection to life-threatening sepsis [1]. Moreover, *S. aureus* evolves various drug resistance mechanisms, subsequently results difficulty in the management of infections. Resistance is, of course, the evolutionary consequence of the deployment of selective pressure; therefore, it has been well indicated among pathogenic bacteria including *S. aureus*. Following discovery of penicillin 1940s, penicillinase producing *S. aureus* were demonstrated, leading to development of semi-synthetic penicillins such as methicillin, which was the most effective antibiotics for penicillin resistant strains [2, 3]. Despite the fact that, the antibiotic was no longer effective due to the emergence of methicillin resistant *S.aureus* (MRSA), which has become a grave public health concern because of higher mortality and morbidity due to invasive systemic infections [4, 5].

Methicillin resistant largely attributed by due to acquisition of mecA gene, found in the Staphylococcal cassette chromosome mec (SCC*mec*) that codes unique penicillin binding protein (PBP2a), which has low affinity to methicillin and other beta-lactam antibiotics. Recently, new resistance gene so called mecC as also identified from MRSA isolates from clinical specimens. Methicillin resistant *Staphylococcus aureus* has mostly considered a nasocomial pathogen since it is increasingly associated with prior exposure to health care facility. Surprisingly, new MRSA variants were also reported from community settings lacking traditional risk factors. Since then, it is well understood that there are two distinct types of MRSA were recognized; hospital-acquired and community-acquired MRSA. Hospital-acquired MRSA strains are resistant not only to beta-lactam agents but also to other types of antibiotics, and mostly associated with type I, II and III SCC*mec*. However, community-acquired MRSA strains are usually resistant to beta-lactams but susceptible to other antimicrobials, and linked mostly with the SCC*mec* type IV and V [6, 7].

According to the evidences, the burden of MRSA has been increasing at an alarming pace throughout the world with showing considerable variation in prevalence according of geographical area or region [8, 9]. Understanding the overall epidemiology of MRSA at country level is so substantial to underpin effective prevention and control strategies. Therefore, the aim of this meta-analysis was to summarize available data and to determine pooled prevalence MRSA and its antibiotic resistance in Ethiopia by conducting a systematic review and meta-analysis.

## Methods

### Study selection

A literature search was conducted in the PubMed, Lancet and Google Scholar databases and articles potentially relevant to our study were identified. The search was performed by six authors (SE, FT, AA, FM, WB and KH) independently, by using the following terms as keywords (and combinations thereof) “*Staphylococcus aureus*”, “*S. aureus*”, “antibiotic resistance profile of *S. aureus*”,” prevalence”, “methicillin resistant *S. aureus*”, “MRSA”, and “Ethiopia”. Among the citations extracted, abstracts were reviewed in an attempt to retrieve the clinical studies on MRSA colonization. Articles that were relevant, by title and abstract, were accessed in full text to determine those that provided sufficient information to be included in our meta-analysis. Finally, the references cited by each eligible study were scrutinized to identify additional articles.

### Inclusion and exclusion criteria

Studies were included in our meta-analysis, if they reported extractable data on the prevalence of MRSA in Ethiopian hospitals or research centers and only English language papers were imposed. On the other hand, studies that did not report on a study of MRSA and failed to comply with Ethiopian setting were excluded from the study.

### Outcome of interest

The major outcome of interest was the prevalence of MRSA among total *S. aureus* clinical isolates. The prevalence was calculated by dividing the numbers of MRSA isolates of the total number of clinically isolated *S. aureus*. As secondary outcomes of interest, we have also calculated the pooled resistance pattern of MRSA isolates to specific antibiotics.

### Data extraction

Data from eligible studies were extracted independently by authors and summarized into a spreadsheet. Discrepancies were resolved by consensus. For each of the included studies, the following information was extracted; name of regions, study area/city, study names, year of the study, study design, types of specimens, numbers of patients/study participants, total numbers of *S. aureus*, proportion of MRSA and references.

### Quality control

The quality of eligible studies was checked independently by three authors (SE, AA, & WB) using a set of predetermined criteria such as research design, quality of paper, and employed methods for MRSA isolation.

### Data analysis

A random effects model was used to determine pooled prevalence and the 95% confidence interval (CI), by employing the approach of DerSimonian and Laird [10]. Besides, Freeman Tukey arcsine methodology was also used to address stabilizing variances [11]. The standard approach of inverse variance method to calculate pooled prevalences does not work well in meta-analysis of single arm study because, for studies with small or large prevalence, the inverse variance method causes the variance becomes small and the calculated CI may outside the range. Therefore, Freeman Tukey arcsine methodology is recommended to correct both variances instability and CIs [12]. We assessed the heterogeneity of study results by the use of I^2^ test. Significant heterogeneity was considered for *P* < 0.10 and I^2^ > 50% [10, 13]. The small study bias was measured by Begg’s funnel plot [14]. The overall prevalence of MRSA was pooled by the forest plot with 95% CI, and regional prevalence was summarized by using figure. In secondary analysis, we have calculated the resistance pattern of MRSA to specific antibiotics, and was presented by using table. Statistical analysis was performed by the use of the Open Meta Analyst (version 3.13) and Comprehensive Meta Analysis (version 3.1).

## Results

**Fig. 1:**
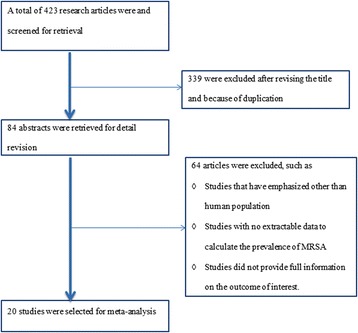
Flow chart shows selected articles for meta-analysis

Through electronic database search, we have found a total of 423 abstracts, among these studies, 321 were disregarded after reviewing their titles, 18 were found to be duplicates, and 64 were excluded because the abstracts or full text information did not directly related to the topic of interest i.e. prevalence of MRSA. Finally, 20 articles fulfilled our eligibility criteria and were subjected to meta-analysis [15–34] (Fig. 1).

**Fig. 2:**
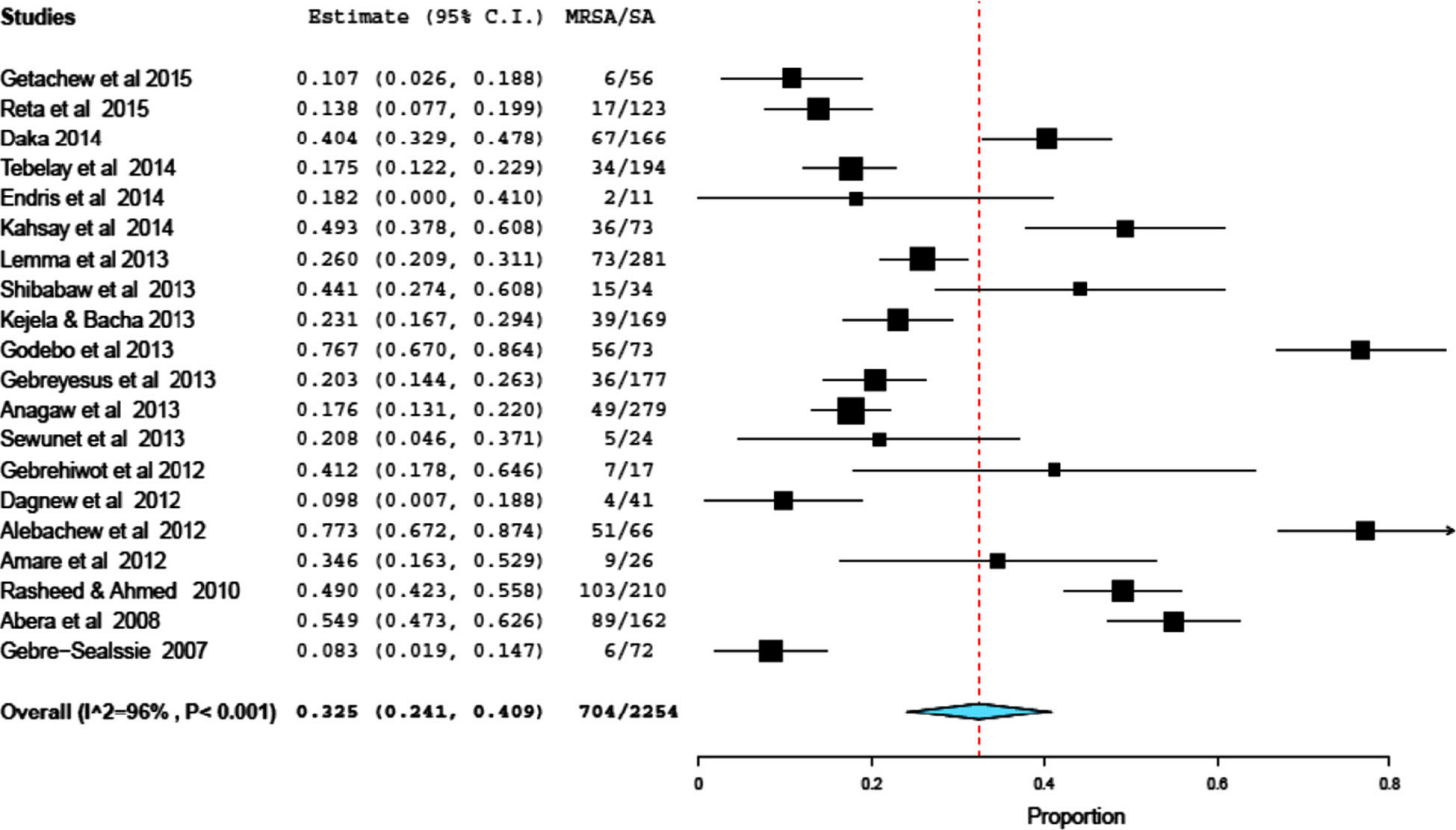
Forest plot of the pooled prevalence of MRSA in 20 studies, Ethiopia, 2004–2015

As presented in Forest plot (Fig. 2), the pooled prevalence of MRSA colonization was 32.5% (95% CI, 24.1 to 40.9%; *P* < 0.001). There was a high level of heterogeneity, random model methods (I^2^ = 96%, *P* <0.001). Since, the included studies have been conducted in different setups, study periods, and study populations, which could have an effect on the heterogeneity of the included studies. Symmetry of funnel plot shows small study bias yielded insignificant effect.

**Fig. 3:**
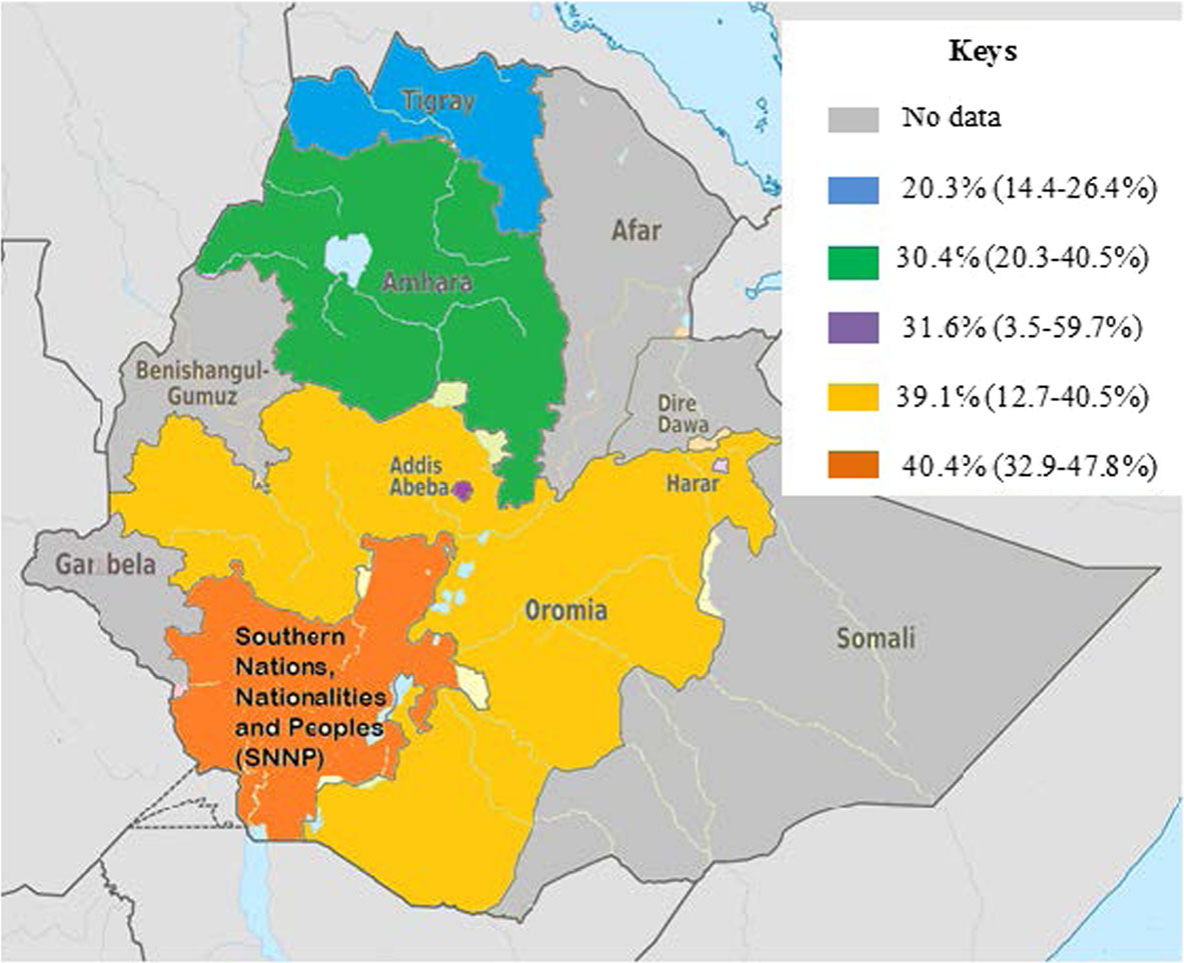
Proportion of MRSA in different regions of Ethiopia, 2004–2015

Selected articles were published from 2007 to 2015 and year of study were ranged from 2004 to 2014. Besides, all included publications were obtained from 4 regions and including the Federal capital city of Ethiopia, Addis Ababa, but no data was obtained from other regions (Afar, Benishangul-Gumuz, Gambela and Somali). Most of the studies indicated that various specimens had been utilized for screening MRSA, especially multisite swabbing was performed from different parts of the body, such as, skin, nasal, eye, ear, urethra, throat, vagina or genital area (Table 1). The lowest and highest proportions of MRSA were reported respectively, from Addis Ababa and Bahirdar cities [30, 33]. As shown from Fig. 3 the average prevalence of MRSA was also noted in different regions of Ethiopia, hence Southern Ethiopia region ranked first (40.4%), followed by Oromia region (39.1%), and Addis Ababa (31.6%), whereas relatively low magnitude of MRSA were demonstrated from Tigray region (20.3%).

**Table 1:** Summary of 20 studies reporting the prevalence of MRSA in different parts of Ethiopia, 2004-2015

Region	Study area	Study period	Study design	Study population	Culture specimens	No of S. aureus isolates	Percentage of MRSA, N (%)	References
Amhara	Bahirdar, Dessie and Debre Tabor	December 2013 to April 2014	Cross-sectional study	HIV infected pediatric patients	Skin swab, nasal swab, and perineum swab	281	73 (26)	Lemma et al. 2015 [17]
	Gondar	February to May 2012	Cross-sectional study	VL patients	Blood	11	2 (18.2)	Endris et al. 2014 [15]
	Gondar	July 2011 to June 2012	Cross-sectional study	Neonates	Blood	17	7 (41.2)	Gebrehiwot et al. 2012 [24]
	Gondar	January to June 2011	Cross-sectional study	Food handlers	Nasal swab	41	4 (9.8)	Dagnew et al. 2012 [25]
	Gondar	September 2009 to June 2010	Cross-sectional study	In and out patient	Urine, eye discharge, genital swab, body fluid, pus, wound swab and discharge	279	49 (17.6)	Anagaw et al. 2013 [22]
	Gondar	January to June 2010	Cross-sectional study	Patients with post-operative surgical site Infections	Pus swab	26	9 (34.6)	Amare et al. 2012 [27]
	Bahirdar	April to June 2006	Cross-sectional study	In and out patient	surgical wound, ear discharges, eye discharges and throat swabs	162	89 (54.9)	Abera et al. 2008 [29]
	Debre Markos	March to June 2013	Cross-sectional study	School children	Nasal swab	123	17 (13.8)	Reta et al. 2015 [12]
	Debre Markos	December 2011 to March 2012	Cross-sectional study	Patients with surgical site infection	Wound swab	73	36 (49.7)	Kahsay et al. 2014 [16]
	Dessie	November 2010 to March 2011	Cross-sectional study	Healthcare workers	Nasal swab	34	15 (44.1)	Shibabaw et al. 2013 [22]
Oromia	Jimma	June to December 2011	Cross-sectional study	In and out patient	Wound swab	73	56 (76.7)	Godebo et al. 2013 [20]
	Jimma	December 2010 to June, 2011	Cross-sectional study	Primary school children and prisoners	Nasal swab	169	39 (23.1)	Kejela and Bacha, 2013 [19]
	Jimma	January 2003 to July 2004	Cross-sectional study	Out patients	Ear discharges, throat and wound swabs	72	6 (8.3)	Gebre-Sealsssie, 2007 [30]
	Harar and Jimma	-	Cross-sectional study	---	Blood, CSF, pus, sputum and urine	210	103 (49)	Rasheed and Ahmed, 2010 [28]
Central Ethiopia	Addis Ababa	March to August 2015	Cross-sectional study	Post operative patients	Wound swab	56	6 (10.7)	Getachew et al. 2015 [11]
	Addis Ababa	September 2013 to April 2014	Cross-sectional study	In and out patient	Nasal swab, wound swab, ear discharge, blood, throat swab, eye swab, vaginal discharge, urethral discharge, urine, stool, sputum, CSF and body fluids	194	34/(17.5)	Tebelay et al. 2014 [14]
	Addis Ababa	March to May 2011	Cross-sectional study	In and out patient (burn patients)	Wound swab	66	51 (77.3)	Alebachew et al. 2012 [26]
	Addis Ababa	April to July 2010	Cross-sectional study	In and out patient (burn patients)	Blood and wound swabs	24	5 (20.8)	Sewunet et al. 2013 [23]
Tigray	Mekelle	November 2010 to January 2011	Cross-sectional study	Health care workers	Nasal swab and material from hand	177	36 (20.3)	Gebreyesus et al. 2013 [21]
Southern Ethiopia	Hawassa	August 2013 to December 2014	Cross-sectional study	Health care workers	Sample from hand and mobile phones	166	67 (40.2)	Daka, 2014 [13]

Keys: *CSF* Cerebro-spinal fluid, *HIV* Human immuno-deficiency virus, *VL* Visceral leshimaniasis

Furthermore, of the selected articles, seven studies have extractable data on the antibiotic resistance profile of MRSA isolates [16, 18, 20, 21, 23, 26, 33]. The pooled resistance rates of MRSA for each tested antibiotics has been presented Table 2; and therefore, high resistant rates were observed to penicillin (99.1, 95% CI: 98–100%), ampicillin (98.1%, 95% CI: 95.7–100%), erythromycin (97.2%, 95% CI: 23.2–100%) and amoxicillin (97.1%, 95% CI: 92.7–100%). In contrast, low level of vancomycin resistance has been calculated, 5.3% (95% CI: 0–10.6%).

**Table 2:** Pooled antibiotic resistance rates of MRSA strains; Ethiopia, 2006-2014

Antibiotics	Antibiotic resistance rate reported by 7 studies							Pooled resistance rate, % (95 % CI)
	Lemma et al. 2015 [17]	Anagaw et al. 2013 [22]	Abera et al. 2008 [29]	Kahsay et al. 2014 [16]	Reta et al. 2015 [12]	Kejela & Bacha, 2013 [19]	Tebelay et al. 2014 [14]	
Chloramphinicol	4 (5.5)	22 (44.9)	-	-	0	23 (59)	16 (47.1)	30.8 (30.3–63.8)
Ceftriaxone	15 (20.5)	2 (4.1)	85 (95.5)	-	0	-		30.8 (0–84.1)
Ciprofloxacin	17 (23.3)	2 (4.1)	67 (75.3)	-	0	-	28 (82.4)	37.3 (5.9–68.7)
Clindamycin	6 (8.2)	1 (2)	-	22 (61.1)	0	-	18 (52.9)	23.1 (7.5–38.8)
Cotrimoxazole	4 (5.5)	22 (44.9)	-	36 (100)	2 (11.8)	7 (17.9)	34 (100)	46.4 (8.4–84.5)
Erythromycin	17 (23.3)	9 (18.4)	69 (77.5)	35 (97.2)	0	24 (61.5)	34 (100)	97.2 (23.2–100)
Tetracycline	53 (72.6)	10 (20.4)	80 (89.9)	15 (41.7)	-	13 (33.3)	-	51.9 (22.8–81)
Pencillin G	-	49 (100)	89 (100)	36 (100)	17 (100)	39 (100)	34 (100)	99.1 (98–100)
Ampicillin	-	46 (93.9)	-	36 (100)	-	39 (100)	-	98.1 (95.7–100)
Amoxacillin	-	46 (93.9)	-	36 (100)	-		-	97.1 (92.7–100)
Vancomycin	-	0	0	-	-	5 (12.8)	10 (29.4)	5.3 (0–10.6)
Gentamycin	-	-	63 (70.8)	34 (94.4)	0	6 (15.4)	13 (38.2)	44.4 (4.9–83.9)

## Discussion

Antibiotic resistance continues to be a global setback in the management of common bacterial infections diseases. Particularly, the problem of antibiotic resistance is highly pronounced in resource limited countries, including Ethiopia, where infectious diseases are rampant [3]. A first global report on the antimicrobial resistance claimed that MRSA is one of the most implicated multidrug resistant (MDR) strains, which has shown a high level of resistance against both beta lactam and non-beta lactam agents [35].

To date, the overall epidemiology and burden of MDR bacteria have not fully understood, especially in resource limited countries [3, 35]. To the best of our knowledge, it is the first a meta-analysis study conducted to determine pooled prevalence of MRSA in Ethiopia. According to this meta-analysis, the overall estimation of MRSA prevalence in Ethiopia was 32.5% (95% CI, 24.1 to 40.9%; *P* < 0.001). This is comparable with meta-analysis studies conducted in similar settings [36–40]. However, this finding is relatively higher than from reports indicated in high income countries [41–43]. It is well known that risk factors associated with MDR strains are comparatively higher in developing countries [3, 44].

Reasonably, there are several issues which can be either directly or indirectly related to the growing burden of MDR prevailing bacterial pathogens including MRSA. Even though, the development of resistance is a normal evolutionary process for microorganisms, but it is highly aggravated by continuous deployment of antimicrobial drugs in treating infections has led to the emergence of resistance among various strains of bacteria [45, 46]. It is claimed that more than 50% of drugs prescribed, sold or dispensed without following standard protocols, and the situation is more magnified in developing countries [47]. In low income countries in particular, antibiotic use is largely relied on clinical judgment without the benefit of specific diagnostic tools, which inevitably leads to rapid evolution of drug resistant strains, which is mainly due to irrational use of antibiotics [48, 49]. Likewise, according to Sosa et al. antibiotic use in most of developing countries is generally unregulated, which is a prime factor for the occurrence of resistant bacterial strains [3]. Therefore, it indicated that antibiotics are widely and inappropriately used in resource limited countries like Ethiopia, resulting to increased prevalence of drug resistant strains such as MRSA.

Additionally, regional prevalence of MRSA was also calculated, hence highest prevalence MRSA (40.4%) was noted in Southern Ethiopia, which was almost two times higher than a result from Tigary region (20.3%). Though, health workers were the study population in the studies of both regions, but as shown in Table 1, different types of samples were collected from participants (health workers), and therefore it would have an effect on the isolation rate of MRSA strains. Besides, the observed variation might be due to different study location, hospital setup, and study period.

Moreover, based on the data have been obtained from seven published articles; MRSA strains showed extremely high resistance rate to penicillin, ampicillin, erythromycin and amoxacillin, in contrast least resistance rate was observed to vancomycin. Similarly, a previous systematic review [50] has also documented that MRSA strains were found to be too highly resistant to the above mentioned antibiotics. It is understood that MRSA strains are able to express beta-lactam hydrolyzing enzymes so called beta-lactamases or capable of modifying penicillin binding proteins [51], hence it is not surprising, MRSA strains are capable of inactivating the beta-lactam agents such as penicillin, ampicillin, cephalosporins, and carbapenems. Even more, MRSA has a propensity to dismantle non-betam lectam agents; this is largely due to co-existence of other resistance gene along with mecA or mecC gene [52]. Most importantly, vancomycin is considerably the most effective and considered as the last resort treatment for resistant infections, especially of MRSA, despite the fact that the emergence of vancomycin resistant organisms has deprived the usefulness of this drug beyond any doubt [53]. Though low levels of vancomycin resistant indicated in this meta-analysis, but it indicates huge blow, especially for the future.

In general based on this finding, the burden of MRSA constitutes a major public health challenge in Ethiopia, thus health care facilities should adopt or establish guidelines to minimize cross-contamination by MRSA. Substantially, maintaining hand hygiene, applying infection prevention protocols, environmental sanitation, and wearing possible personal protective equipment are advocated for preventing infection. In addition to that promoting health education, professional educations as well as public awareness campaigns are evidently effective in the reduction of the unnecessary use of antibiotics, which in turn reduce selective pressure of mutant strains.

## Conclusion

In this meta-analysis the pooled MRSA prevalence is considerably high. Aside from that more than 97% of MRSA strain was found to be highly resistant to beta-lactam agents (penicillin, ampicillin and amoxacillin) and non-beta-lactam agents (erythromycin). In contrary, low level of resistance rate was observed to vancomycin. Thus, to combat the burden of MRSA in particular, the following concerns should be considered at the national level, such as adopting safety protocols and implementing proper antibiotic prescription policies.

## Abbreviations

CI: Confidence interval
MDR: Multidrug resistant
MRSA: Methicillin resistant *Staphylococcus aureus*
SCC*mec*: Staphylococcal cassette chromosome mec.
